# Mapping resistance responses to *Sclerotinia* infestation in introgression lines of *Brassica juncea* carrying genomic segments from wild *Brassicaceae B*. *fruticulosa*

**DOI:** 10.1038/s41598-017-05992-9

**Published:** 2017-07-19

**Authors:** Kusum Rana, Chhaya Atri, Mehak Gupta, Javed Akhatar, Prabhjodh S. Sandhu, Nitin Kumar, Ravinder Jaswal, Martin J. Barbetti, Surinder S. Banga

**Affiliations:** 1 DBT Centre of Excellence on Brassicas, Department of Plant Breeding and Genetics, Punjab Agricultural University, Ludhiana, 141004 Punjab India; 2 School of Agriculture and Environment and the UWA Institute of Agriculture, Faculty of Science, The University of Western Australia, 35 Stirling Highway, Crawley, WA 6009 Australia

## Abstract

Sclerotinia stem rot (*Sclerotinia sclerotiorum*) is a major disease of *Brassica* oilseeds. As suitable donors to develop resistant cultivars are not available in crop Brassicas, we introgressed resistance from a wild *Brassicaceae* species, *B*. *fruticulosa*. We produced 206 *B*. *juncea*-*B*. *fruticulosa* introgression lines (ILs). These were assessed for pollen grain fertility, genome size variations and resistance responses to *Sclerotinia* following stem inoculations under disease-conducive conditions. Of these, 115 ILs showing normal fertility and genome size were selected for cytogenetic characterization using florescent genomic *in situ* hybridization (Fl-GISH). *B*. *fruticulosa* segment substitutions were indicated in 28 ILs. These were predominantly terminal and located on B-genome chromosomes. A final set of 93 highly fertile and euploid (2n = 36) ILs were repeat-evaluated for their resistance responses during 2014–15. They were also genotyped with 202 transferable and 60 candidate gene SSRs. Association mapping allowed detection of ten significant marker trait associations (MTAs) after Bonferroni correction. These were: CNU-m157-2, RA2G05, CNU-m353-3, CNU-m442-5, ACMP00454-2, ACMP00454-3, EIN2-3-1, M641-1, Na10D09-1 and Na10D11-1. This is the first time such a molecular mapping technique has been deployed with introgression lines carrying genomic segments from *B*. *fruticulosa*, and the first to show that they possess high levels of resistance against *S*. *sclerotiorum*.

## Introduction

Indian mustard (*Brassica juncea*) is an important winter season oilseed crop in India. It contributes nearly 28% of edible oil supplies there^1^. *B*. *juncea* is also cultivated in China and eastern Europe as oilseed, vegetable or condiment crop(s)^2^. With the development of canola quality *B*. *juncea*, possibilities exist for its further expansion to drier regions in Australia and Canada^3^.While enhancing yield remains a major breeding objective, protecting yield potential against disease threats is equally important. Such threats to *B*. *juncea* include diseases such as sclerotinia stem rot, alternaria blight, white rust and downy mildew. Of these, sclerotinia stem rot, caused by *Sclerotinia sclerotiorum* ^4^, is now a major threat to the continued sustainability of this crop across the mustard growing belt of India^5–7^. In severe cases, incidence of sclerotinia stem rot can be up to 92%^8–10^. Sclerotinia stem rot is also now recognized as a major yield limiting factor for *B*. *napus* in Canada, Europe, Australia and China^11–15^.

*S*. *sclerotiorum* has a vast host range worldwide^16, 17^. While no commercial crop *Brassica* varieties have been bred specifically for resistance to sclerotinia stem rot, some *B*. *napus* varieties with increased tolerance to this disease have been released in China^18, 19^. However, available level of resistance within the crop Brassicas is insufficient as a source to meet the level of resistance needed to effectively combat this pathogen in commercial varieties. Identifying sources of resistance in *Brassica* has been challenging due to variations in plant screening assays utilized and varied aggressiveness of *S*. *sclerotiorum* isolates^20–22^.

Studies on genetics of resistance show a complex picture of being either monogenic and/or polygenic depending on the plant species and material under investigation^23–26^. Quantitative trait loci (QTL) mapping method for polygenic resistance is helping to identify loci related to *S*. *sclerotiorum* resistance in several crop species like soybean, common bean, sunflower and *B*. *napus* ^27–33^. Several of the resistance-related QTLs were found to be associated with A (A02, A03, A09) or C (C02, C04, C06, C07, C09) genomes of *B*. *napus*. A, B and C genomes are associated with *B*. *rapa*, *B*. *nigra* and *B*. *oleracea* species, respectively^34–36^. Comparative mapping analysis with *Arabidopsis thaliana* further showed a candidate gene, *BnaC*.*IGMT5*, was concomitant with a major QTL SRC6 in *B*. *napus*. Co-localization of four QTLs for field resistance with three flowering time QTLs within an 8.1-cM region on the linkage group C02 was also reported^37^. However, the phenotypic variation explained by such QTLs is generally very small like in *B*. *napus*, ranging between 4–10% and 6–22%. Furthermore, Wei *et al*.^37^ identified 6 and 5 QTLs for Sclerotinia resistance in field and controlled conditions, respectively, and 17 QTLs for flowering time in *B*. *napus* ^38^. Identification of QTLs with such a low level of phenotypic variation can be attributed to a low-moderate level of resistance in the populations/parents used for mapping. In QTL mapping studies, there can be issues related to limited recombination events, low allele number and the need for longer research time. Association mapping is now rated highly in terms of mapping resolution, allele number and time-saving in establishing marker trait associations^39, 40^. However, there are very few reports of association mapping in *B*. *juncea* ^41^, despite its extensive usage for characterizing resistance to blackleg stem canker caused by *Leptosphaeria maculans* and for establishing genetic diversity and LD in 192 inbred lines by using 451 single locus SSRs^42, 43^. Separate QTLs for leaf and stem resistance against *S*. *sclerotiorum* have been reported in *B*. *olerace*a^44^. In other crops, like soybean, Iquira *et al*. genotyped and mapped 3 QTLs against Sclerotinia stem rot by GBS approach^45^.

Although, the taxonomic family *Brassicaceae* (*Cruciferae*) represents a huge pool of genetic diversity, only a few species such as *Erucastrum gallicum*, *Capsella bursa*-*pastoris*, *E*. *cardaminoides*, *D*. *tenuisiliqua* and *B*. *fruticulosa* and *B*. *oleracea* have been reported to carry high levels of resistance against this pathogen^46–50^. While there is only one report regarding introgression of genomic segments responsible for resistance from the wild species *E*. *cardaminoides* or *D*. *tenuisiliqua* into the cultivated *B*. *juncea*, subsequently it was also possible to introgress *B*. *fruticulosa* resistance into *B*. *juncea* (S. S. Banga, unpublished). In this communication, we report molecular characterization of *B*. *juncea*-*B*. *fruticulosa* introgression lines (ILs) using molecular cytogenetic studies and a set of transferable and candidate gene based SSR markers. We also report resistance-associated QTLs and the underlying candidate genes against *S*. *sclerotiorum*. This is the first time such a molecular mapping technique has been deployed with ILs carrying genomic segments from wild *Brassicaceae* species possessing high levels of resistance against *S*. *sclerotiorum*.

## Results

### Pollen grain fertility and genome size variations

**Figure 1:**
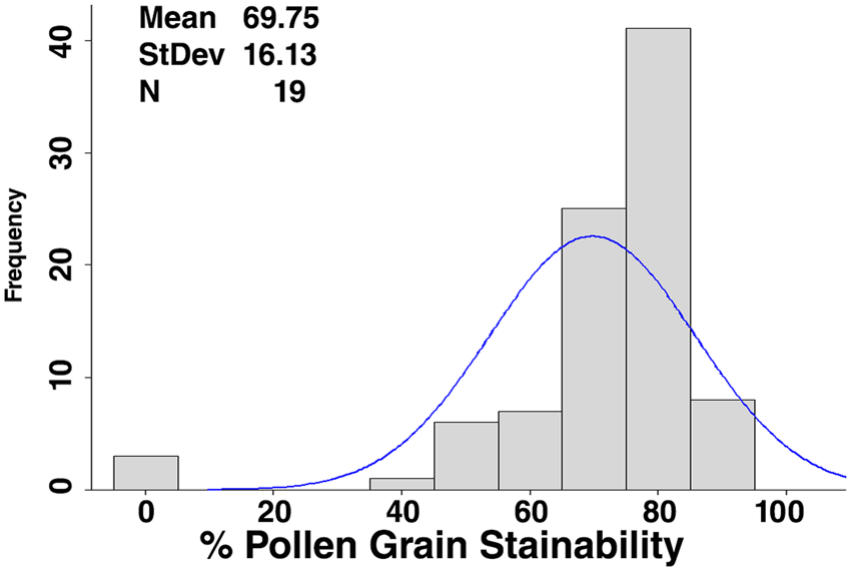
Pollen grain fertility of introgression lines, showing variation from 40% to 90%.

**Figure 2:**
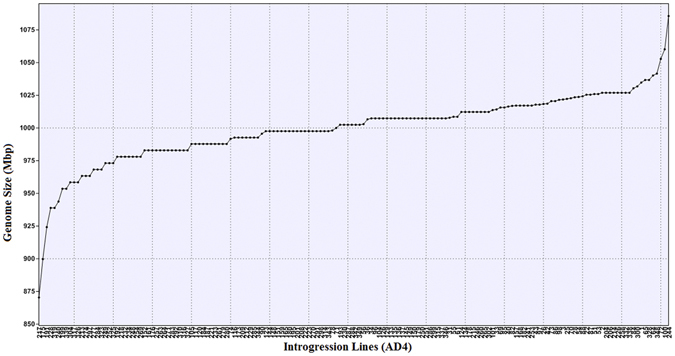
Variation in genome size between introgression lines. Genome size varied from 870.42 to 1085.58 Mbp. Genotype RLC-1 had an average genome size of 1045.482 Mbp.

Pollen grain fertility in the introgression set (AD-4), comprising 206 ILs, varied from 40% (IL-251) to 90% (IL-152, IL-215, IL-332) with a relatively greater number of genotypes clustering around 75% pollen fertility (Fig. 1). Twenty four lines showed intermediate pollen grain fertility. The estimation of DNA content in cell nuclei of *B*. *juncea*-*B*. *fruticulosa* ILs showed significant genome size variations (SE 2.08, CV 2.66). Generally, the 1C DNA content in the ILs varied from 870.42–1085.58 Mbp (Fig. 2). Natural *B*. *juncea* genotype, RLC-1 has an average genome size of 1045.482 Mbp. The largest genome size was found in IL-244 (1085.58 Mbp) followed by IL-100 (1060.152 Mbp). IL-217 (870.42 Mbp) had the smallest genome size.

### *In situ*–hybridization

**Figure 3:**
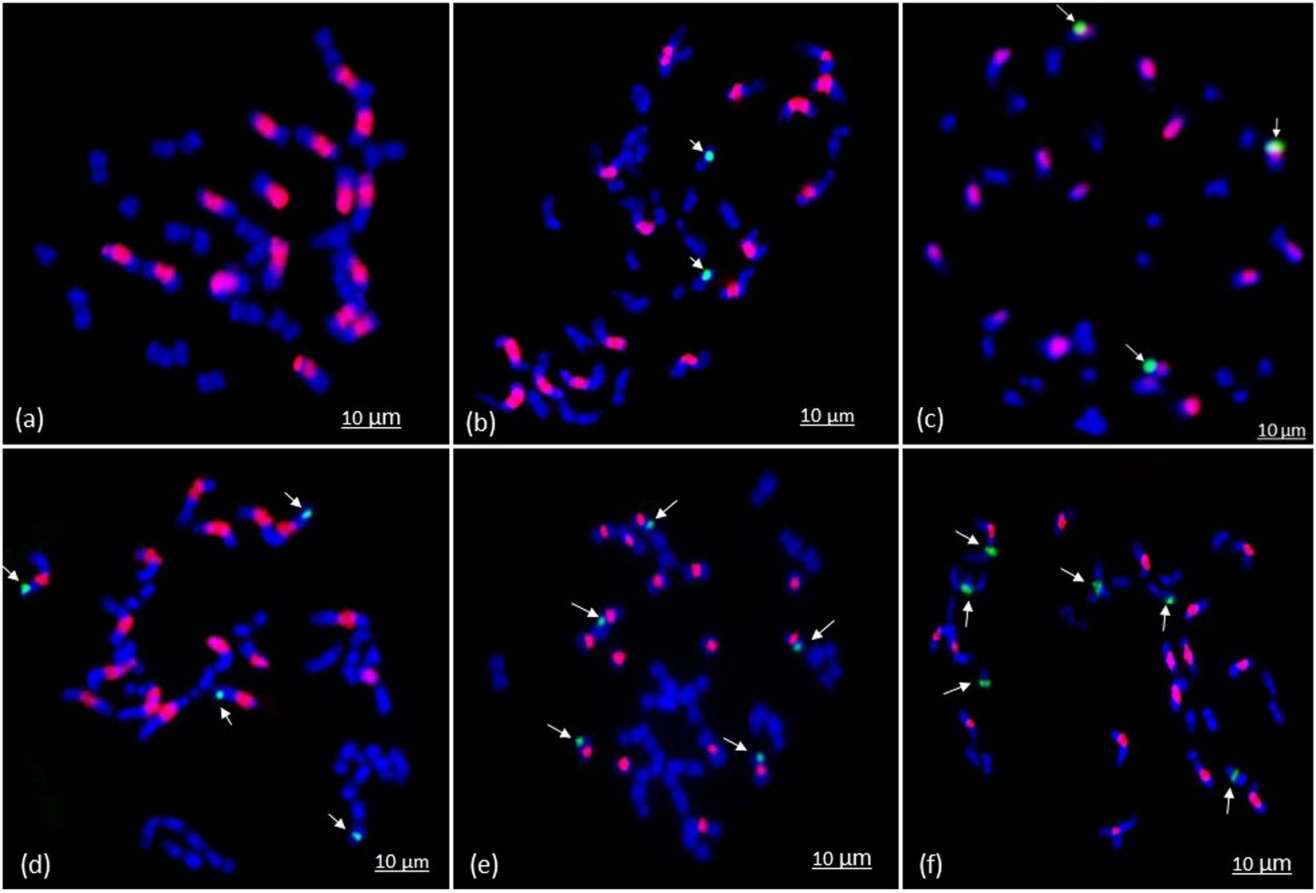
Genomic *in situ* hybridization on mitotic spreads of introgression lines (ILs) of *B*. *juncea* (AABB). B genome is painted in red while *B*. *fruticulosa* introgressions are shown in green colour. (**A**) *B*. *juncea* with no introgression, (**B**) IL with two *fruticulosa* segment substitutions in the A-genome, (**C**) IL with three *fruticulosa* segment substitutions in the B-genome, (**D**) IL with three *fruticulosa* segment substitutions in the B-genome and one in A-genome, (**E**) IL with five *fruticulosa* segment substitutions in the B-genome, (**F**) IL with six *fruticulosa* segment substitutions in the A-genome.

One hundred and fifteen *B*. *juncea*-*B*. *fruticulosa* ILs with high pollen fertility and near normal genome size were selected for intensive molecular cytogenetic investigations. These were checked for chromosome number and also explored for the presence of *B*. *fruticulosa* introgression(s) using *B*. *fruticulosa* specific probes. We also used *B*. *nigra* probes to differentiate between A- or B-genomes of *B*. *juncea*, found to be harbouring *fruticulosa* introgressions. Out of 115 ILs investigated, 108 had normal euploid chromosome number (2n = 36). Of these, 28 ILs revealed large *B*. *fruticulosa* chromosome fragment substitutions. Segment substitutions were recorded on 2–6 recipient chromosomes in 28 ILs (Fig. 3A–F). Number and size of such alien chromosome fragment substitutions varied. Simultaneous staining with *B*. *nigra* specific probes revealed that B-genome chromosomes of *B*. *juncea* harboured 83.84% of the identified *B*. *fruticulosa* chromosome segment substitutions. Alien fragment substitutions were primarily terminal. Only a small fraction of introgressed fragments were located on intercalary positions. These showed normal chromosome pairing during meiosis.

### Disease Expression

**Figure 4:**
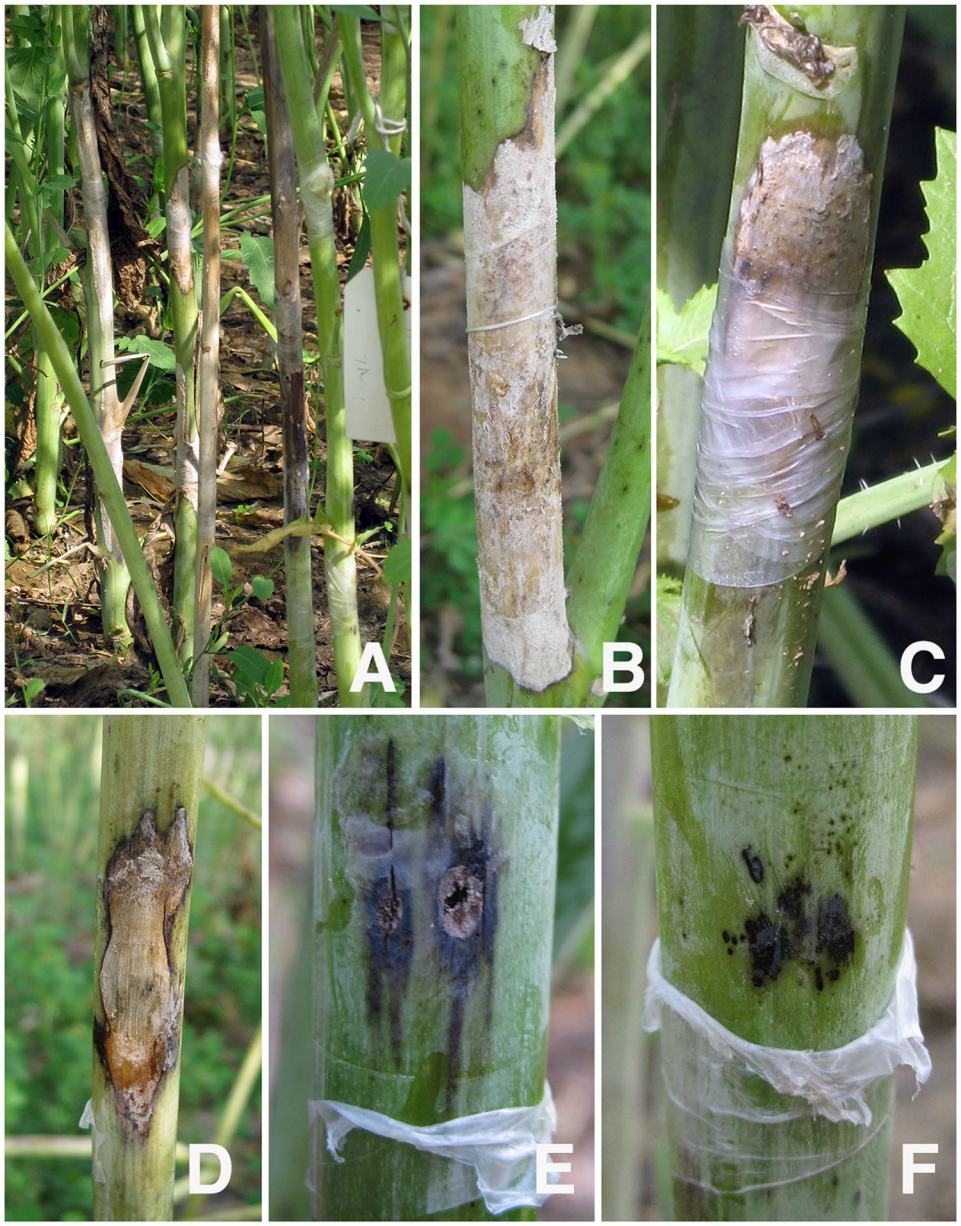
Resistance expression of introgression lines against *Sclerotinia sclerotiorum*. Introgression lines were grouped into five resistance categories. (**A**) Highly susceptible, (**B**) susceptible, (**C**) moderately resistant, (**D**) resistant, (**E** and **F**) hypersensitive resistance response; with stem lesion lengths ranging from >10.0; 7.5 to 10.0; 5.0 to <7.5; 2.5 to <5.0; and <2.5 cm, respectively.

**Figure 5:**
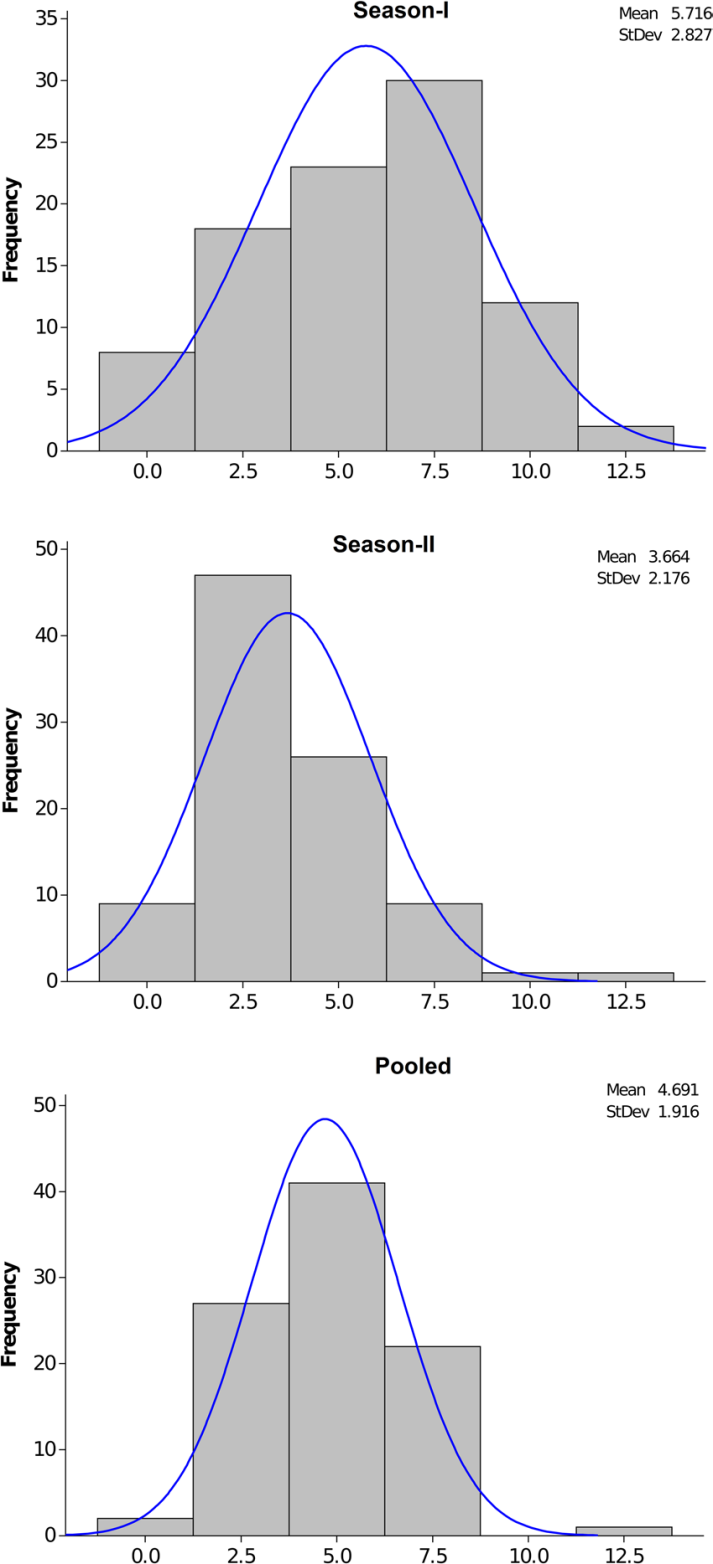
The logarithmic values (% proportion) for 93 *B*. *juncea*-*B*. *fruticulosa* introgression lines in season I (2012–2013), season II (2013–2014), and pooled across both seasons I and II, as against K values. Histogram shows frequency of ILs in season I and season II against *Sclerotinia sclerotiorum*.

We first evaluated an extended set of 206 ILs along with resistant donor (*B*. *fruticulosa*) and susceptible recipient (*B*. *juncea* cv. RLC 1) parents for their responses to stem inoculation during 2011–12. Of these, a smaller set of 93 ILs were selected on the basis of high fertility and euploid chromosome number. These were repeat evaluated during 2014–15 for their responses to stem inoculation. Significant (P < 0.05) variations were indicated among the ILs in terms of expression of resistance responses to stem inoculation. Test genotypes were classified between highly susceptible to highly resistant classes (Fig. 4). Results were largely consistent over two crop seasons (Fig. 5). The LS mean value for stem lesion length in season I (SI) was 5.72 cm and for season II (SII) it was 3.66 cm. The corresponding values for the susceptible parent RLC-1 and resistant *B*. *fruticulosa* were 11.5, 11.75 and 0.62, 0.32 cm, during seasons I and II, respectively. Of the ILs evaluated, 38 and 65 ILs fell under the resistant category during two crop seasons. Among these, 13 genotypes showed a highly resistant response in season I and 29 genotypes in season II, with a mean lesion length less than 2.5 cm. Dunnet test established them to be significantly superior than the susceptible recipient, RLC 1. Frequency histogram revealed a marginal overrepresentation of the resistant class (Fig. 5). The analysis of variance revealed significant genotypic differences between the test ILs for their resistance responses to stem inoculation.

### Genetic diversity, population structure, linkage disequilibrium

**Figure 6:**
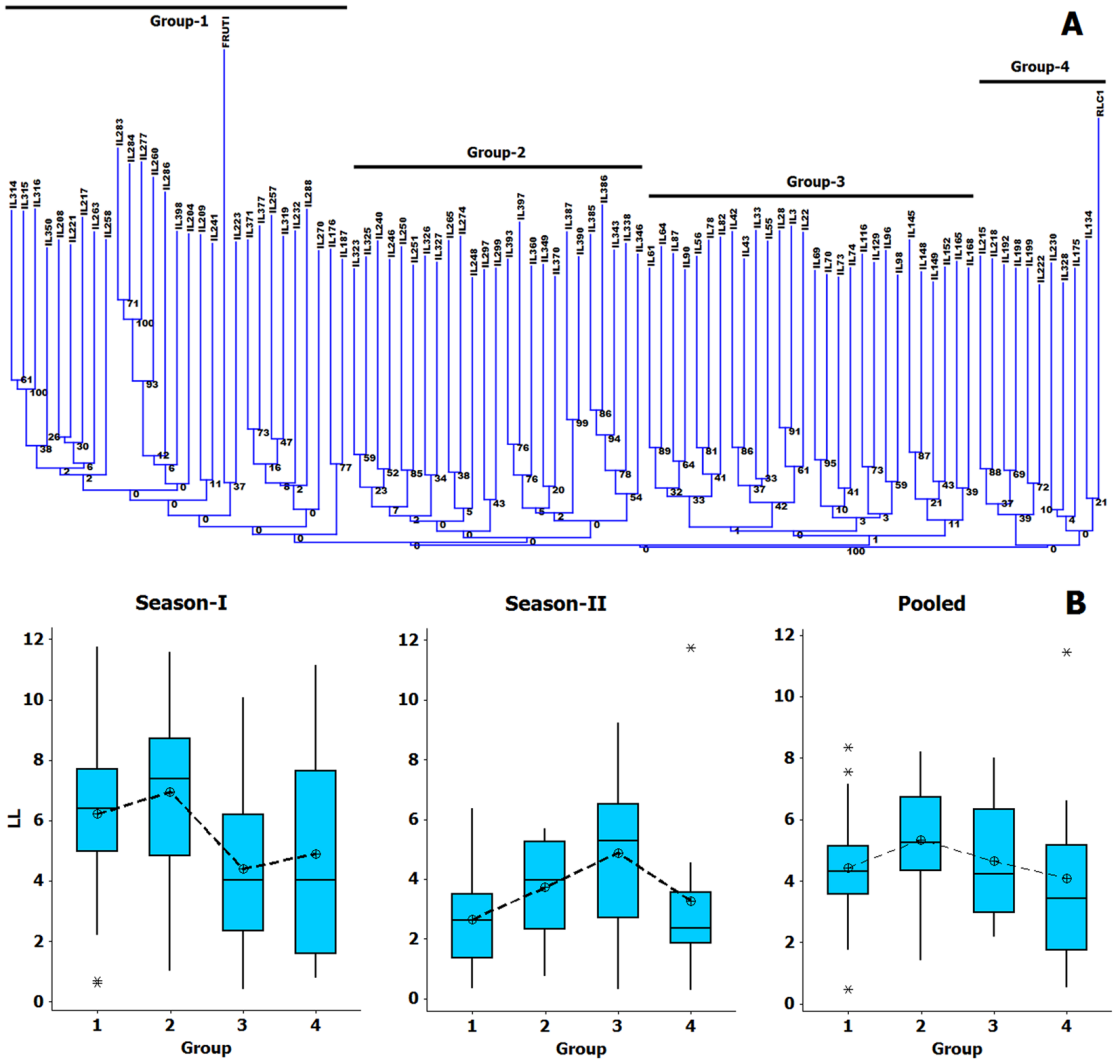
(**A**) Dendrogram based on Gower’s similarity index showing patterns of genetic variation in introgression lines. (**B**) Box plots showing average performance of introgression lines grouped in a given cluster.

ILs along with two parents were genotyped with a set of 262 SSRs. These included 202 transferable SSRs, obtained from A- or B-genomes of crop *Brassicas*. Remaining 60 SSRs were developed on the basis of sequence information from 14 candidate genes, reportedly associated with sclerotinia resistance. These markers amplified 509 alleles. DNA polymorphism data were then used to establish genetic diversity in the given set of ILs. Diversity tree based on the SSR markers formed four clusters on the basis of Gower’s similarity coefficient (Fig. 6A). These four clusters included 29, 25, 27 and 12 genotypes, respectively. Wild donor species *B*. *fruticulosa* was included in cluster one, while the recipient *B*. *juncea* parent RLC 1 was present in cluster four. In terms of resistant genotypes, clusters three and four included maximum proportion(s) of resistant genotypes (Fig. 6B).

**Figure 7:**
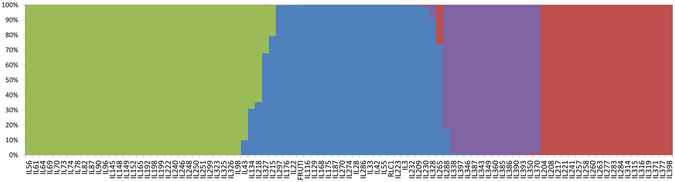
Population structure of introgression lines based on 262 markers. The K is at 4 as the introgression lines clearly differentiated into three distinct clusters.

As the genetic structure of the mapping panel is important for association mapping, we used STRUCTURE software to deduce the groups or clusters based on the average LnP(D) values. ∆K appeared maximum at K = 4, dividing the population into four distinct clusters, epitomized by different colors (Fig. 7). A total of 34 ILs was included in the cluster I, whereas clusters II, III and IV comprised of 26, 14 and 19 ILs, respectively. Most of the resistant and highly resistant introgression lines were included in population groups I and II (<4.0 LS-mean stem lesion length in pooled data). These groups are represented by green and blue colors respectively. In contrast groups III and IV constituted a mixture of moderately resistant, susceptible and highly susceptible lines.

### Association mapping

LS-means of phenotypic data were used for association studies. Of the four different models considered, GLM (P3) was the best fitted model, having observed P-values closest to the expected P values. Linkage disequilibrium (LD) was evaluated for global markers as well as significant markers in SI, SII and data pooled over two crop seasons. The R^2^ values of SSR markers were plotted against genetic distances. The R^2^ values of global SSRs in SI and SII were 0.013406 and 0.015688, respectively. For significant marker loci, R^2^ values were 0.1054 and 0.1241, respectively. Significant (P < 0.0001) LD blocks were observed for SSR markers EIN2-2, CNUm157, CNUm442,CNU_m353, NA10011, NA10009, RA2G05 (Supplementary Fig. S1). The markers with −log10 P value > 3.0 after Bonferroni correction were considered significantly associated with *S*. *sclerotiorum* resistance.

A total of 10 significant marker trait associations were detected across two seasons (Table 1). Of these, 5 marker loci were A or B-genome specific, 5 were with unknown marker positions. One marker loci, RAG2G05, exhibited association in season I as well as on the basis of data pooled over two seasons, having −log10P values varying from 3.01 to 3.45. This marker explained 10.0 to 13.06% of phenotypic variation, respectively. Other significantly associated markers were CNU_m353, ACMP00454 and M6341. These had −log10P values varying from 3.01 to 4.08 as observed during season II as well as for data pooled over two seasons. Phenotypic variation explained by these significant marker loci ranged from 10.51 to 15.28%. CNU_m157, Cnu_m442, EIN2-3, m6341, Na10D09 and Na10D11 were also involved in significant markers-trait-associations. Gene prediction analyses established the biological relevance of these trait markers associations as follows: CNU_m353 appeared associated with major intrinsic protein Aquaporine like (NIP3-1) and methyl transferase activity; Na_10_D_11_was co-related with cysteine type peptidase activity (Ulp-1protease family); and ACMP00454 seemed concomitant with the defensive AT hook motif nuclear localized protein.

**Table 1:** Marker-Traits-Associations of SSR molecular markers against *Sclerotinia* stem rot.

Marker loci	Chrm.	Chrm. Position (cM)	Season I		Season II		Pooled	
			RsqMarker^b^	−Log10^a^	RsqMarker^b^	−Log 10^a^	RsqMarker^b^	−Log 10^a^
CNU-m157-2	A9	51.8	0.1108	3.31	—	—	—	—
RA2G05	—	—	0.1000	3.01	—	—	0.1306	3.45
CNU-m353-3	A5	42.2	—	—	0.1468	3.37	0.1478	3.15
CNU-m442-5	A5	79.1	—		0.1159	3.29	—	—
ACMP00454-2	A9	—	—	—	0.1181	3.35	0.1528	4.00
ACMP00454-3	A9	—	—	—	0.1459	4.08	0.1447	3.79
EIN2-3_1	—	—	—	—	0.1382	3.15	—	—
M634-1	—	—	—	—	0.1051	3.01	0.1147	3.06
Na10D09-1	—	—	—	—	0.1176	3.34	—	—
Na10D11-1	—	—	—	—	0.1058	3.03	—	—

^a^Negative logarithm value of *P* value of each associated marker.

^b^Phenotypic variance explained by each associated marker (%).

## Discussion

Introgressive breeding is an important technique to introduce novel traits into the cultivated forms. It is especially consequential, if the genetic variation for the desired trait is not available in the primary germplasm. *B*. *juncea* is generally highly susceptible to *S*. *sclerotiorum*. There is only one previous report demonstrating introgression of genomic segments responsible for resistance against *S*. *sclerotiorum* from the wild species *E*. *cardaminoides* or *D*. *tenuisiliqua* into the cultivated *B*. *juncea* ^49^ and a single unpublished study demonstrating successful introgression of *B*. *fruticulosa* resistance into *B*. *juncea* (S. S. Banga, unpublished). Advanced generation BC_1_S_5_ ILs, selected for the present studies were fully fertile and had euploid chromosome number. These were developed in the genetic background of a recipient *B*. *juncea* cv. RLC 1. The presence of a uniform background in ILs with different and homozygous introgressed donor segments allowed us to establish differences among the introgressed and native alleles. Molecular markers have been used in the past for mapping populations derived from distantly related crosses and many QTLs were identified^51^. IL analysis uses lines fixed for each QTL in the background of an elite breeding line, and can produce acceptable results. Molecular cytogenetic (Fl-GISH) coupled with molecular mapping technique helped to identify ILs carrying genomic segments from *B*. *fruticulosa*. These possessed high levels of resistance against *S*. *sclerotiorum*. Resistance-associated QTLs and the underlying candidates in introgression lines were successfully aligned with their resistance responses to stem inoculation with *S*. *sclerotiorum*. Ten significant marker trait associations were detected, of which five involved A-genome marker loci. The present study not only provides important new insights into the resistance mechanisms within *B*. *juncea*-*B*. *fruticulosa* introgression lines against *S*. *sclerotiorum*, but opens the way for novel engineering of new *B*. *juncea* varieties that express resistance that is critical to enabling better management of this worldwide devastating pathogen of rapeseed-mustard crops.

GISH studies involving *B*. *juncea*-*B*. *fruticulosa* introgression lines confirmed large *B*. *fruticulosa* introgression or segment substitution in 28 lines. These were predominantly terminal and ranged from 2–6 chromosome fragment substitution per IL. However, it was not possible to estimate exact size of introgressed fragments as *Brassica* chromosomes are very small and there were cell to cell variations for chromosome condensation patterns. Allosyndetic pairing, chromosome breakages and reunions are known to precipitate multiple translocations leading to introgression of genomic fragments from wild into crop genomes. Most of such chromosome fragment substitutions are expected to be terminal as these require only one break while intercalary fragment exchanges necessitates two breaks. Their location on B-genome chromosomes of *B*. *juncea* was probably due to strong homoeology that exist between *B*. *nigra* and *B*. *fruticulosa*; both belong to the same *Sinapis* lineage^52–54^. Random introgressions of uncharacterized DNA segments from unadapted wild germplasm are also likely to have structural and/or functional consequences for the recipient genome. This was apparent from low pollen grain fertility and genome size variations in many *B*. *juncea*-*B*. *fruticulosa* ILs carrying alien chromosomal fragments. Such variations primarily result from loss or gain of chromatin during initial generations following wide hybridization^54, 55^. The observed DNA changes were not simply unidirectional as upward as well as the downward shift in the genome size was indicated in the present study. However, average 1C genome size (1008.561 Mbp) of the ILs was very close to the genome size of natural *B*. *juncea* (1045.482 Mbp).

As expected in an advanced generation of backcross selfs, the ILs were phenotypically homogeneous, as introgressed genomic variation is expected to become stabilized and also Mendelized after many generations of selfing. While variation in pollen fertility observed in ILs may result from the genomic rearrangements expected during inter-specific hybridization, male sterility was the likely outcome of nucleo-cytoplasmic interaction between *B*. *juncea* and *B*. *fruticulosa* cytoplasm^56, 57^. Resistance responses of a subset of 91 ILs to *S*. *sclerotiorum* infestations revealed excellent variation for the trait investigated. These also suggested that several *B*. *fruticulosa* chromosome segments may affect resistance responses, and imply that gene(s) for resistance could be characterized and associated to specific marker alleles. Near normal distribution of resistance responses pointed towards a quantitative inheritance. Group memberships varied between clustering and population structure patterns. Such variations are expected because clustering is based on empirical groupings by similarity values and bootstrapping constancy whereas structure is model based, assuming the loci are at Hardy-Weinberg equilibrium and linkage equilibrium within populations.

Association mapping studies revealed that significant MTAs could explain up to 30% of the phenotypic variation in our introgression set, which possibly explained only a part of the available variation for resistance. In terms of biological relevance of identified MTAs: CNU_m353 could be linked to major intrinsic protein Aquaporine like (NIP3-1) and methyl transferase activity. This confirms a previous report suggesting its association with methyl-transferase activity^58^. ACMP00454 was related with the defensive AT hook motif nuclear localized protein. The AT hook motif nuclear localized protein is known to be involved in resistance against bacterial and oomycete pathogens^59^. AT hook motif containing transcription factor (CaAtl1) is associated with the defense response in transgenic tomato plants^60^. MTA involving EIN2-2 was detected only during the season II. The EIN-like proteins are known to be involved in regulation of biotic, abiotic and oxidative stresses, disease resistances and in regulation of ethylene responsive genes^61^. Combined use of genomic SSRs and candidate gene based SSRs can be a useful strategy, especially in situations where genomic resources from wild donor species are not available. While genome-wide association mapping surveys the genetic variation in the whole genome and locates signals of association for complex traits^62^, the candidate gene-based association mapping (CG-AM) allows a more focused approach and requires prior knowledge about gene associations involving a target phenotype^63, 64^. Extent of associations with identified markers underlined their role in defining introgressed resistance to the stem rot.

Summarizing, we believe that Sclerotinia resistance can be influenced by many uncharacterized genes contributing to overall phenotypic expression, as is commonly observed for quantitative traits. However, results from association mapping studies clearly demonstrated that *B*. *juncea*-*B*. *fruticulosa* ILs have significant potential breeding applications. This offers mustard breeders a powerful tool to optimize use of the genetic variation available within wild *Brassicaceae* by bringing together in one genotype the different alleles that maximize resistance to *S*. *sclerotiorum* for future mustard cultivars. Such novel engineering of new *B*. *juncea* varieties that express these critical resistances to *S*. *sclerotiorum* will enable more effective management of this worldwide devastating pathogen of mustard crops.

## Methods

### Plant materials

*B*. *juncea*-*B*. *fruticulosa* introgression set was developed by first hybridizing wild crucifer *B*. *fruticulosa* (2n = 16) with *B*. *rapa* (2n = 20). The synthetic amphiploid (FFAA; 2n = 36) was subsequently backcrossed twice with *B*. *juncea* (RLC-1) and advanced by following single seed descent method to develop a BC_1_S_5–6_ populations.

### Pollen grain fertility

Percent pollen fertility was determined by staining pollen grains from freshly dehisced anther(s) in acetocarmine (2%) solution. For each line, >100 pollen grains were scored in different microscopic fields. Intensely stained and normal shaped pollen grains were scored as fertile while those unstained and/or collapsed were scored as sterile.

### Nuclear DNA content

The DNA content was estimated by using Flow cytometry to determine the genome size variations. A reagent kit, PartecCyStain UV precise P, was used for nuclei extraction and DNA staining of nuclear DNA from plant tissues^65^. The method involves preparation of aqueous suspensions of intact nuclei by placing about 0.5 cm^2^ of young leaf tissue in a Petri dish and adding 400 µl of extraction buffer. The sample was finely chopped and filtered through a Partec 50 µm Cell Trics disposable filter into the flow cuvette. Staining solution (1.6 ml) was added to this supernatant and allowed to incubate at room temperature for 60 sec. The resultant sample was then analyzed with PartecCyFlowPloidy Analyzer with UV-laser excitation. A suspension of nuclei of CRBCs (chicken red blood cells) was used for calibration with PC-5 (*B*. *carinata*) as an internal reference standard. The DNA content of reference sample was calculated based on tomato *Lycopersicon esculentum* genotype “*Stupickepolnityckoverane”*, as an external reference standard. It had a 2C content of 1.96 pg. Two replications per each genotype were sampled for ploidy screening. The absolute DNA content of a sample was calculated based on the given formula where 1 pg corresponded to 0.978*10^9^ bp^66, 67^. Reference genome size of *Brassica juncea* (http://www.Brassica.info/info/reference/genomesize.php) was used for comparison.

### Chromosome preparation

To prepare mitotic slides, roots were first harvested from two days old germinating seeds raised on moist filter paper at 20–25 °C. Harvested roots (=30 mm) were treated with saturated aqueous solution of α-bromonaphthalene for 4 h, followed by fixation in alcohol: acetic acid (3: l) for at least 2 h at 4 °C. To prepare roots for *in*-*situ* hybridizations, these were first washed in a citrate buffer for 30 min to remove the fixative and subsequently incubated in the enzyme mixture containing 2% (w/v) cellulase and 20% (v/v) pectinase in 4 mmol citrate buffer (Citric acid monohydrate and trisodium citrate dehydrate), pH 4.8 for about 1 h at 37 °C. Treated roots were agitated with a micropipette tip to discharge mitotic cells in the micro-centrifuge tubes. These were centrifuged for 3 min at 600–800 g, followed by 45 min treatment in KCl (150 mmol) for 20 min. The cells were washed thrice at 800 g for 3 min in freshly prepared fixative to clear the cytoplasm. One drop of 7 µl of suspension was released on acid cleaned chilled slide from a height of 50 mm to spread the cells on the slide. Slides were air-dried in a desiccator before further use.

### Probe preparation and *in*-*situ* hybridization

Purified DNA of *B*. *fruticulosa*, *B*. *nigra* and *B*. *juncea* were extracted using the DNeasy plant mini kit (Qiagen) according to manufacturer’s instructions. To prepare the probes, genomic DNA of *B*. *fruticulosa* was labelled with fluorescein-12-dUTPandgenomic and DNA of *B*. *nigra* was labelled with tetramethyl rhodamine-5-dUTP, using a nick translation kit (Roche, Germany) according to manufacturer instruction. GISH was performed in two step hybridization process. For first *in situ* hybridization, 40 μl of hybridization mixture containing 50% formamide, 2x SSC, 10% dextran sulphate, 0.025 μg salmon sperm DNA, 1.25 mM EDTA, 0.125% SDS, 200 ng of labelled *B*. *fruticulosa* probe and 100 fold *B*. *juncea* blocking DNA was applied on slides with good chromosome spreads. This was followed by incubation at 80 °C for 4 min in a thermocycler. These slides were kept in the hybridization chamber overnight at 37 °C. Slides were then washed at 42 °C for 2 min in 2x SSC and 5 min in 0.1x SSC (three times), respectively, and cooled to room temperature in 2x SSC for 5 min. After that, slides were incubated in 4x SSC in 1% tween-20 for 5 min. The chromosomes were stained in Vectashield mounting medium containing DAPI. The chromosome preparations were reused for the second GISH detection with *B*. *nigra* whole genomic probe without blocking DNA to identify genome specific introgression. Visualization was performed with Zeiss fluorescent microscope (ImagerZ2 AX10). Digital images were captured using Isis^®^ software. Images were cropped and optimized using only functions affecting the whole image with Image J.

### DNA extraction and SSR genotyping

Genomic DNA was harvested from young leaves using standard procedures for DNA extraction^68^ 93 ILs, along with parents. Parental polymorphism assays were carried out using over 800 SSR primers, representing all the 18 *B*. *juncea* chromosomes. Of these, only 202 SSRs [164 markers for developed from A-genome^69^ and 38 from B-genome (sequences obtained on MTA from Isobel Parkin, Canada)] were polymorphic. PCR amplification of these markers was positive for *B*. *fruticulosa* but negative for recipient *B*. *juncea* parent. Fourteen candidate genes for *S*. *sclerotiorum* resistance in *Arabidopsis thaliana* ^70, 71^ were also used in this study. These included: SAH (S-adenosyl-L homocysteinase), CYP450 (Cytochrome P450), OMT (O-methyltransferase), Photosystem II polypeptide, MYB (Myeloblastosis), COI1 (Coronatine Insensitive 1), IGMT-5 (Indole glucosinolate methyl-transferase 5), NPR1 (Nonexpresser of Pr genes 1), EIN2 (Ethylene Insensitive 2), ABI1 (ABA Insensitive 1), ABI2 (ABA Insensitive 2), DET3 (De-etiolated 3), PAD3 (Phytoalexin Deficient 3) and LACS2 (long-Chain Acyl-CoA Synthetase 2)^72, 73^. The nucleotide sequences of identified defensive genes were retrieved from the TAIR (Arabidopsis Information Resource) database. The syntenic regions from *A*. *thaliana* genome were used to search for similar regions in *B*. *rapa* using nucleotide–nucleotide BLAST (blastn) algorithm^74^. Overlapping primers were designed for identified genes using BLAST tool provided by National Centre for Biotechnology Information (http://www.ncbi.nlm.nih.gov/tools/primer-blast/). Primer3 web software was used to design primers and perform BLASTN search in the selected database to check the specificity of the primers. The primers were designed with optimum melting temperature between 50–60 °C with GC content ranging from 30–60%. Product size varied from 250 to 500 bp. Designed primers were tested for specificity, amplification and optimum annealing temperature on the genomic DNA of both parents, RLC-1 and *B*. *fruticulosa*. The list of candidate genes, TAIR accession number along with gene functions and primers developed are presented in Tables S2 and S3. The amplification for total 262 polymorphic makers was carried out in a final volume of 10.33 µl, containing 25 ng of template DNA, primers; 1 µM each forward and reverse, dNTPs- 400 µm, 1 X PCR buffer with MgCl_2_ and Taq DNA polymerase 1 unit. The PCR reactions were performed in a 384 well Applied Biosystems thermocycler (Model no. EN61328). The amplified DNA product was fractioned using an automated high-throughput electrophoresis system (Caliper Lab Chip GX version 3.0.618.0). Allelic polymorphism of all the markers was recorded and mapping position was inferred from published data^75^. Markers with more than 25% of missing data were not used for further analysis.

### Disease assessment

A set of 206 BC_1_S_5_ *B*. *juncea* genotypes were initially evaluated for their resistance response against *S*. *sclerotiorum*. The test genotypes were sown in a randomized complete block design with two replications during the 2011–12 winter growing season. Of these, a set of 93 genotypes (BC_1_S_6_) were selected on the basis of high pollen grain stainability, euploid chromosome number, normal meiosis and disease response. These were repeat-evaluated during 2014–15 winter growing season, in a randomized complete block design with two replications. Each test genotype was raised in 3 m long paired rows with a row-row spacing of 30 cm. High humidity was maintained by using adequately placed foggers (1 fogger/9 m^2^). Foggers are operated 2–3 times a day for 15 min or more, depending upon the prevailing weather conditions.

### *Sclerotinia sclerotiorum* inoculum

Sclerotia of *S*. *sclerotiorum* isolate PAU-4, collected from infested *B*. *juncea* fields, were surface sterilized in 50% (v/v) sodium hypochlorite and 70% ethanol, each for 4 min, followed by two washes in sterile distilled water for 1 min^76^. The isolate was cultured on potato dextrose agar medium at 20 °C with 12/12 h light/dark. The daughter sclerotia were harvested from the incubated plates after four weeks and maintained at 4 °C for subsequent inoculations. The relative resistance responses across ILs were evaluated using a modified stem inoculation method^77, 78^. Ten random plants in the middle of each row were selected for inoculation when 50% of the plants in the row had achieved flowering. Each selected plant was inoculated by placing a single *S*. *sclerotiorum* mycelial plug disc (5–8 mm diameter), cut from the actively growing margin of a 48 h culture grown on a glucose-rich medium containing peptone (peptone 10 g, glucose 20 g, agar 18 g, KH_2_PO_4_ 0.5 g, H_2_O 1 L, pH 6.0 before autoclaving), onto the stem immediately above the first node and fixing with Parafilm^®^. Stem lesion lengths were measured with a linear ruler after three weeks of inoculation as described previously^79, 80^. ILs were grouped into five resistance categories, viz. highly resistant (HR), resistant (R), moderately resistant (MR), susceptible (S) and highly susceptible (HS) with stem lesion lengths ranging from 0 to <2.5; 2.5 to <5.0; 5.0 to <7.5; 7.5 to 10.0 and >10.0 cm, respectively^54^. RLC-1 and *B*. *fruticulosa* were used as susceptible and resistant checks, respectively. LS-means of lesion length were calculated by PB Tools (http://bbi.irri.org/) for two seasons. Values were also pooled over seasons to determine appropriately adjusted means for the other effects in the model. Boxplot and Dunnett’s test method were used in ANOVA to create confidence intervals in pooled values for differences between the adjusted mean of each introgression line and highly susceptible genotype RLC-1 as a control. The test was carried out with family error rate (0.05) for all comparisons. Dunnett’s method determines the confidence levels for each individual comparison accordingly.

### Genetic diversity, population structure and marker-trait-association

Paleontological statistics software package (PAST) was used to establish genetic diversity^81^ in the introgression set. We estimated the proportion of donor genome introgression in the ILs from the number of SSR markers. We calculated the marker-based value by dividing the total number of SSR markers for the donor genotype that were present in the ILs by the total number of SSR markers used for the SSR genotyping. The Bayesian model based program STRUCTURE 2.2 was used to infer the population structure and K inferred as number of populations^82^. Real number of clusters was inferred through an *ad*-*hoc* measure of K based on relative change in LnP (D) between successive K^83^. The software STRUCTURE Harvester was used to estimate the actual number of K. For association mapping^84^, resistance responses in terms of lesion length were standardized by representing lesion length of the test genotype as a proportion of the lesion length of susceptible check. Adjusted mean value lesion length data were used for downstream analysis, using four different models [GLM + K3, MLM + K3), GLM + PC3 and MLM + PC3] as implemented in TASSEL V 2.1^85^. Q and PC were used as model covariates to reduce false associations. The Bonferroni correction threshold was calculated for 262 markers at 5% level of significance. As the probabilities were too small for analysis, they are converted into −log10P values. MEGANTE (https://megante.dna.affrc.go.jp/) was used for gene prediction analyses to establish biological relevance of identified MTAs.

## Electronic supplementary material

Supplementary information
