# Supplementary material for: Mapping resistance responses to Sclerotinia infestation in introgression lines of Brassica juncea carrying genomic segments from wild Brassicaceae B. fruticulosa
Source: Sci Rep. 2017 Jul 19;7:5904. doi: 10.1038/s41598-017-05992-9 (PMC5517529; doi:10.1038/s41598-017-05992-9)
Supplement: Supplementary file 1 — Supplementary information [file 41598_2017_5992_MOESM1_ESM.pdf]

Mapping resistance responses to *Sclerotinia* infestation in introgression lines of *Brassica juncea* carrying genomic segments from wild *Brassicaceae B. fruticulosa*

Kusum Rana, Chhaya Atri, **Mehak Gupta, Javed Akhatar, Prabhjodh S. Sandhu**, **Nitin Kumar**, **Ravinder Jaswal**, DBT Centre of Excellence on Brassicas, Department of Plant Breeding and Genetics, Punjab Agricultural University, Ludhiana, 141004 Punjab, India; **Martin J. Barbetti**, School of Agriculture and Environment and the UWA Institute of Agriculture, Faculty of Science, The University of Western Australia, 35 Stirling Highway, Crawley, WA, 6009, Australia; and **Surinder S. Banga***, DBT Centre of Excellence on Brassicas, Department of Plant Breeding and Genetics, Punjab Agricultural University, Ludhiana, 141004 Punjab, India.

*Corresponding author: S. S. Banga; E-mail address: nppbg@pau.edu

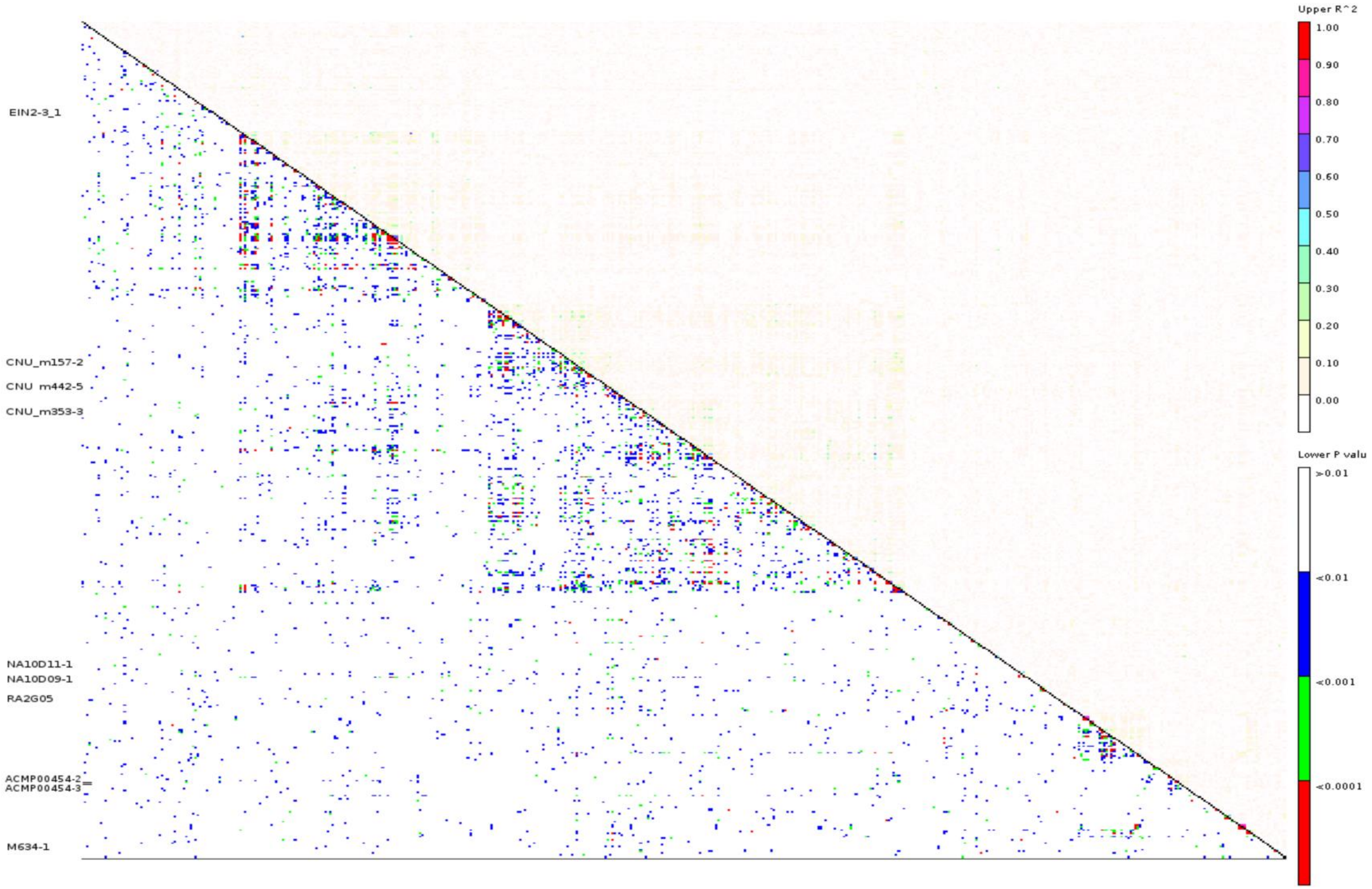

Supplementary figure S1: The r^2 values of SSR markers plotted against genetic distances using software TASSEL V 2.1. The r^2 values of global SSRs in season I and season II were 0.013406 and 0.015688, respectively ($P < 0.0001$).

Supplementary Table S1. *Arabidopsis thaliana* defense related genes, TAIR accession numbers, *Brassica* candidate genes and gene functions.

Candidate Genes	TAIR accession number[a]	*Brassica* candidate genes[b]	Functions
IGMT5	AT1G76790	BNIGMT5	Related with SRC6 locus and reported for *Sclerotinia* resistance
OMT1	AT1G21110	BROMT1	Regulatory role in development, reproduction and defense system interaction with environment factors
NPR1	AT1G64280	BJNPR1	Controls the onset of SAR (systemic acquired resistance) and resistance response against broad spectrum of pathogens
ABI1	AT4G26080	BJABI1	Involved in ABA signalling pathway and has function in responses to wounding and stress
ABI2	AT5G57050	BJABI2	
SAH2	AT3G23810	BRSAH2	Involved in genomoic methylation of pathogens, key enzyme in transmethylation reactions and resistance against several pathogens
PAD3	AT3G26830	BRPAD3	Encodes an enzyme required for camalexin biosynthesis, involved in resistance to fungal pathogens
MYB	AT1G79180	BJMYB	Characterized by R2R3 type MYB domain, involved in development, signal transduction, disease resistance and abiotic stress tolerance
COL1	AT2G39940	BRCOI1	Involved in jasmonate regulated defense against necrotrophic pathogens, wound healing. Consists of LRR and F-box motif
CYP450	AT4G22690	BRCYP450	Mediates synthesis of primary and secondary phytoalexins and alkaloids
DET3	AT1G12840	BRDET3	Reveals central role for the vacuolar ion exchange ATPase in plant growth and development, role in cell elongation and meristem activity
EIN2	AT5G03280	BREIN2	Involved in regulation of oxidative stress, high salt concentration, disease resistance and regulation of ethylene responsive genes
LACS2	AT1G49430	BNLACS2	Involved in cutin biosynthesis, plays crucial role in tolerance to biotic and abiotic stresses
PSII	AT1G79040	BJPSII	Encodes 9.3 KD alpha subunit and 4.3 KD beta subunit, involved in electron transport reactions

[a] TAIR: The Arabidopsis Information Resource (http://arabidopsis.org)
[b] Candidate genes names were given with BN (*B. napus*), BJ (*B. juncea*) and BR (*B. rapa*) prefix as designed from the species.

Supplementary Table S2. List of candidate gene specific primers used in candidate gene based association mapping

Genes	Primer	Forward sequence	Reverse sequence	Amplicon Size (bp)
**NPR1**	BJnpr 1-1	TTTGAAACACACCTGCAGCT	TCGGAGTAGACTAGAAGCGC	420
	BJnpr 1-2	GCGCTTCTAGTCTACTCCGA	GTTGACAAAAGGGGCAAACG	449
	BJnpr 1-3	CGTTTGCCCCTTTTGTCAAC	GGAGGTTCTCTACGTGGCTT	560
	BJnpr 1-4	AATCTGGAGGACGAAAGCCA	GGTGGATGTATCGGCGTTAC	421
	BJnpr 1-5	CATCGGCGAGAATCAGCTTG	TGATTGCTCCTTGTTCGTAGA	281
**ABI1**	BJABI1-1	ATGGAGGAAGTATCTCCGGC	AATGAGCGGTTGACTGAGGA	451
	BJABI1-2	GGTCGGTTCAATCCTCAGTC	TGGACAAAGGAAGGGCAGTT	450
	BJABI1-3	AACTGCCCTTCCTTTGTCCA	AGGGTTTGCTCTTGAATTTCCT	660
**ABI2**	BJABI2-1	ATGGACAAAGCTTCTCCTGC	CGAGATGATGATGAAGTAGAGCC	200
	BJABI2-2	CGCCGGAGAAGGGATCAA	CTGCCACGTGTCGCCATC	474
	BJABI2-3	TGAGGAGATAGCCAGGGAGA	TGGATCTTGACATGGCGAGA	452
	BJABI2-4	GCTGGAGGGAAAGTGATCAG	AGGGTTTGCTCTTGAATTTCCT	495
**COI 1**	BRCOI1-1	TCCGGATATCAAGAAGTGCAGA	CTGCAGTGCTTAACGATGCT	480
	BRCOI1-2	TCAGCATCGTTAAGCACTGC	TGTCGAGAAAACAGAAGAAGCA	738
	BRCOI1-3	GTAACTGGCTTCATGAACTTGC	CAGCACTGTCCAAGAACCTC	600
	BRCOI1-4	ATTGGAGATAGGGGTCTAGAGGT	ACAACCTCTCATCTCCAGCT	510
	BRCOI1-5	GGATGTCCGAAACTACAGAAGC	TCATCATCATCATGCCTCCCT	330
	BRCOI1-6	TGCTGTTGTATTGCCAATGGT	CACACAGTTCATGGTAACCCC	263
**EIN2**	BREIN2-1	GCATATCGCGACAATCTGAAAG	CAACCCTTGACCCCAAATTAGA	276
	BREIN2-2	GGGGTCAAGGGTTGGAGTAG	AGGAGCGTTGTTGTTGTTGT	226
	BREIN2-3	ACAACAACAACAACGCTCCT	CAAACGCAACCATCTCCACA	262
**PAD3**	BRPAD3-1	ACCAAGAAAACAGGACAACGA	CTGAAGAGCTCGATAACAGCG	421
	BRPAD3-2	TAGGGTTCATGCCTTACGGT	TGGTCGGCTTGAAAGAGTCT	543
	BRPAD3-3	TGAGGAATCAAGACAAAGATGGA	TTCTGGATCACGTGCAATCG	464
	BRPAD3-4	CGATTGCACGTGATCCAGAA	GTGGTGAAGAACTTGAAAGAGGT	281
**DET3**	BRDET3-1	GGCTACCTGAGACTGAATATTGT	TTTGTGGAATGAGTTTTGCAGA	295
**LACS2**	BNLACS2-1	AGCTGCTGATCATGTGGTG	ACCTTAGAATCAACCGTTACGC	213
	BNLACS2-2	TGAAGCAATATCCGAATGAGCA	GGTACATAAGTTATCCCTTGGCT	235
	BNLACS2-3	GCCAAGGGATAACTTATGTACCT	ACATTGGTTCTCAGCTTCTTCC	215
	BNLACS2-4	TCGACTACTCAAAAGGAAGAAGC	CCACGCCAGTATCCAACAGA	350

	BNLACS2-5	GACATTCGGTACTTGATGGATGA	TGAGAGAGATTAGAGGCAGGG	341
	BNLACS2-6	TGAGAATCATCCCTGCCTCT	GAACCATCCATTGATCACGACT	281
	BNLACS2-7	ACCACAAAAGACAAGACCTAACT	TGGTATTGCTTTGCGGTAGAG	385
	BNLACS2-8	GCTTAACTCTACCGCAAAGCA	TCCGGTTCTATGCTCTGGAC	203
**IGMT**	BNIGMT-5-1	AGTGAACTCAATGACCCACAA	TGACACAAGCACCTTCTTATCC	469
	BNIGMT-5-2	TTCTACAAACATATCTCCAGCCA	TGATTTTACCGACTTGCTCAAAC	430
	BNIGMT-5-3	ATTGGTTTGAGCAAGTCGGT	ACTCGACAGTGTCTTCCTCA	460
	BNIGMT-5-4	GACACTGTCGAGTTCAAGAATGA	ACGTTTCAGACCCTAAATCCATG	419
**OMT**	BROMT1-1	AGAGAACTCAATGACCCACAAA	TTGGAGTGATGAAGACTGTGC	280
	BROMT1-2	ACAGTCTTCATCACTCCAATCA	TGTGGTGCTAGAAGGAGGAG	431
	BROMT1-3	ACATCTTTCAGTTGTGCCCTG	ACTCGACAGTGTCTTCCTGA	565
	BROMT1-4	GACACTGTCGAGTTCAAGAATGA	TGGAGATGATGAGACCGAGC	385
**SAHH2**	BRSAH2-1	AGCCTCTCAAAACCAAGAACA	GGACTTGAGACCTTCCCTGG	546
	BRSAH2-2	TCAAAGTGGCCAATGTTGCA	CCAAAACACGGTTGAAGTTGT	447
	BRSAH2-3	ACAGAAACCCAAAACACTAAACC	TTCGGTTTTATTAGGGGAAGACA	463
	BRSAH2-4	TGTCTTCCCCTAATAAAACCGAA	CGGTGGTGATGCTACTCTCT	468
	BRSAH2-5	ACGATCAGATCAGGACCACC	TTGTGGAGAAGACGTCGAGT	400
**CYP450**	BRCYP450-1	AGGAACCTTGCACATAGGGG	CGGACTCGCTATGCATAAGAG	267
	BRCYP450-2	TGCAAGGACGAGGTGATCT	GGAGACAACCCTAATGAGTTCA	273
	BRCYP450-3	GCGGCTGTGTCACGAGAAA	AGTTAGGAATGTGGTCGGTGA	222
	BRCYP450-4	TTGTCACCGACCACATTCCT	CCTAAGCCTGAGACGGAGAG	230
	BRCYP450-5	CTCTCCGTCTCAGGCTTAGG	ACCAACTGTGTCGTCTGTAGA	269
	BRCYP450-6	TCTACAGACGACACAGTTGGT	GTCGTGAGCTTGGTTTCGG	221
	BRCYP450-7	CTGTCCGAAACCAAGCTCAC	TCTTCTTGATTATTGCCGCGA	344
**PSII PP**	BJPSII-1	GGGAGAACATAGAGATCGAGTC	TGAGCGACGGTAATCCTCTC	151
	BJPSII-2	CACAAGAGCTTCTCCTTCTTCC	TTGTAAACACCGTATCCCTTTCC	150
	BJPSII-3	AGTACGGTGCTAACGTCGAT	ACATAGAACAGAACAACGACACT	240
**MYB**	BJMYB-1	GAGAGAGGGAATGGGGAAGG	TCCAACACAAGAAGCATGCA	403
	BJMYB-2	TGCATGCTTCTTGTGTTGGA	TGAATAATGTCATTAAACGCGCT	363
	BJMYB-3	AGCGCGTTTAATGACATTATTCA	TGAAGATGGTTTAGTCTCAGAGC	473
